# Supplementary figures and images for: Pancreatic Cancer Cell-Derived Exosomes Promote Lymphangiogenesis by Downregulating ABHD11-AS1 Expression
Source: Cancers (Basel). 2022 Sep 23;14(19):4612. doi: 10.3390/cancers14194612 (PMC9562033; doi:10.3390/cancers14194612)

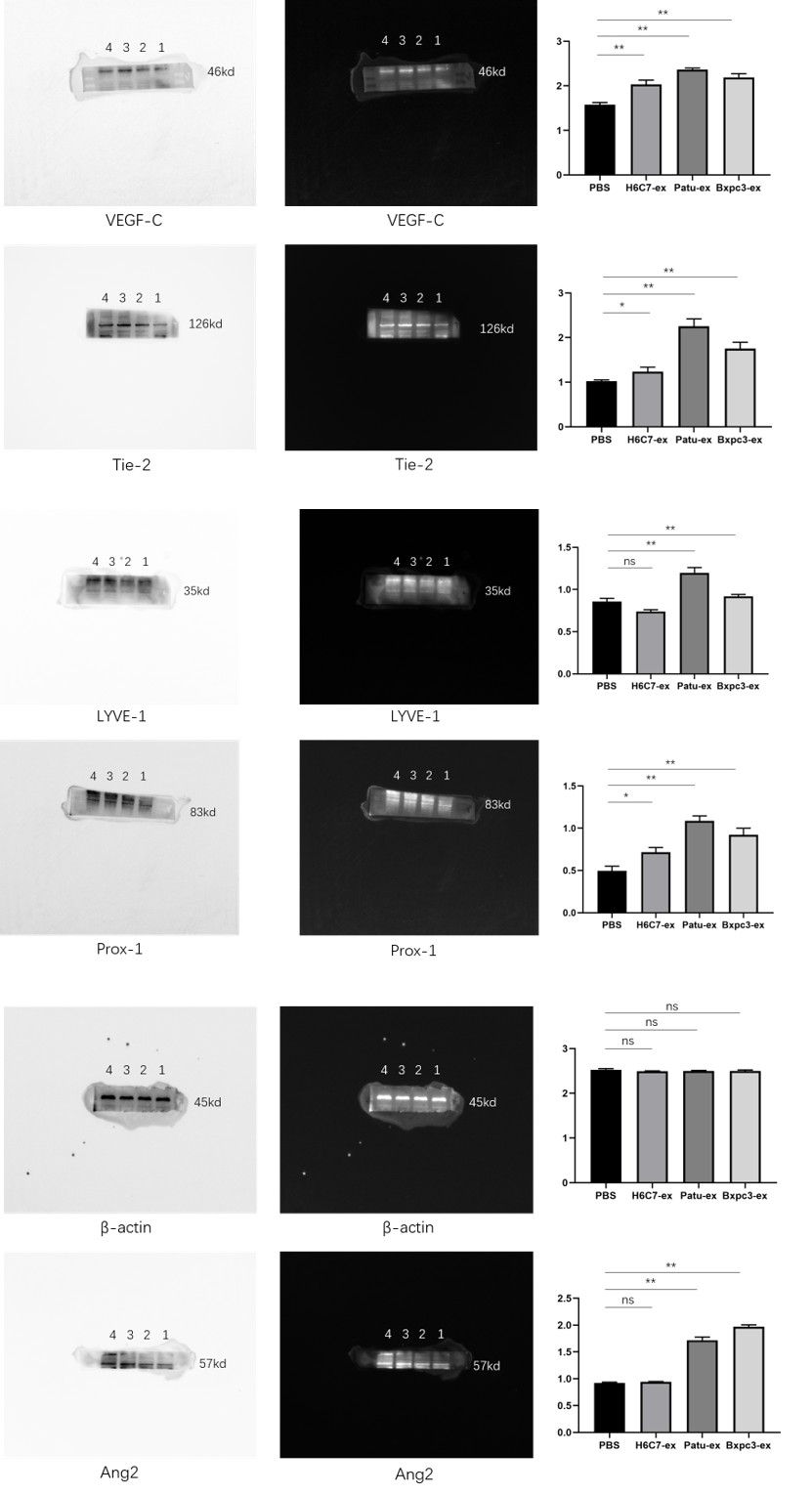

Supplement: Supplementary file 1 [file cancers-14-04612-s001.zip › cancers-1869968-supplementary.jpg]
